# Selections

**Published:** 1897-10

**Authors:** 


### Selections.

### ACETANILIDE AS A DRESSING.

MORTON writes in the *Polyclinic* of February 16, 1895, that the action of acetanilide upon wounds, especially granulations, when used in full strength, is to produce intense dryness, blueness, and to check at once and to prevent the formation of pus. Upon extensive granulating surfaces and chronic ulcers a slight burning sensation is at first perceived, which is rapidly succeeded by a sedative or anæsthetic effect. If used in sufficient quantity, a thin scab of acetanilide, combined with the wound secretions, forms, under which healing rapidly progresses. If a very large surface is exposed to the action of the undiluted drug, toxic symptoms promptly supervene in susceptible individuals. It is probable that children and the aged are more sensitive to its absorption than are vigorous middle-aged persons. It is also probable that anæmia might follow too prolonged application of large quantities of the substance, because of its destructive action upon the red blood-corpuscles; this, however, he has not seen. The powder does not, as a rule, stick to wounds or hold dressings fast; but when it does so, alcohol causes instant release by dissolving the drug.

Under no circumstances does acetanilide irritate the skin or wounds, even when used beneath impervious protectives or antiseptic poultices.

What may be the best vehicles for applying acetanilide must yet be proved. Upon most of his cases the pure powder was used from a dusting-box. This, while usually safe, he thinks has been an unnecessary waste, for very recent experiments in dilution have shown him that a one-fifth- to one-per-cent. mixture with petrolatum was sufficient to arrest suppuration and secure rapid healing in an extensive septic scald. All pain vanished after the first application.

Acetanilide dissolves in five volumes of alcohol, in twenty volumes of ether, and in two hundred volumes of water. It is soluble in liquid petrolatum to the extent of forty grains to the ounce. In chloroform it very freely dissolves.

By diluting with water a saturated alcoholic solution of acetanilide, the drug will be thrown out of solution in the shape of fine crystals, and will remain perfectly mixed in suspension long enough to permit of its use in this form as an injection for abscesses or carbuncles, in gonorrhœa, etc.

In the large number of cases in which he has freely employed acetanilide, but twice have toxic effects been noticed; one was in an infant aged fourteen months.—*The Therapeutic Gazette.*

---

### COCAINE.

At the meeting of the Académie de Médecine, M. Reclus made some remarks anent cocaine. He said that local anæsthesia by injections of cocaine, of which he has been a strong partisan for the last ten years, has not yet found many adherents. He thought it necessary, consequently, to recall in a few words the principles to be observed in its employment, and to explain at the same time the reason why accidents have been reported from time to time from its use. At first he would affirm once more the eminent anæsthetic properties of cocaine, which he considered to be superior to all those used with the same therapeutic object, and in particular to guaiacol, that had recently been warmly recommended by one of his colleagues.

He (the speaker) had made a comparative study of both of these agents, employing them in the region he was about to operate, one on either side; he found that anæsthesia was complete in the part which had received the injection of cocaine, while the sensibility was not entirely abolished in the region submitted to the guaiacol. The accidents attributed to cocaine were due to the operator, and not to the drug, and could have been easily avoided if the indications he had repeatedly laid down had been followed, and which consisted in employing only one-per-cent. solutions, and never to exceed the total dose of three or four grains of cocaine, to always place the patient in a recumbent position, and to avoid penetrating a vein.

It was by observing these rules that he was able to practise three thousand five hundred operations without one accident; not even did he once observe an attack of syncope or vomiting.

He employed cocaine exclusively in cases where the field of operation was not too extensive. That is to say, he did not use it in abdominal surgery nor in amputations of the limbs. However, in two cases, where he was not able to give chloroform by reason of cardiac trouble, he used with success cocaine in amputating the arm.—*Paris Cor. Med. Press and Circular.*

---

### UROTROPINE.

The favorable reports that have come to my notice regarding urotropin lead me to think that some additional facts concerning it may not be amiss. Urotropine is a derivative of formic aldehyde, and not a coal-tar product. The well-known antiseptic and preservative power of formic aldehyde in solution led Nicolaier, of Göttingen, to use the solution of formic aldehyde, containing forty per cent. of the gas, and known as formalin, as a means of preserving specimens of urine from decomposition pending examination. He noticed that neither uric acid nor the amorphous urates were deposited, though the same specimens not treated with formalin deposited either or both, as the case might be. Even when already precipitated, these substances underwent solution when formic aldehyde was added to the specimen. Urines that readily deposited large amounts of uric acid when acidulated with a mineral acid did not do so when a sufficient amount of the preservative was added.

The results of these investigations prove very conclusively the great uric acid solvent power of formalin. The reputation of the lithium salts as uric acid solvents is caused by the common error of applying the results of laboratory experiments to practical therapeutics. The fact that lithium forms an insoluble phosphate, and that the blood and urine contain considerable amounts of soluble phosphates, show that lithium salts, given by the mouth, must form insoluble phosphates of lithium long before they can combine with the uric acid. In this respect it is no exception to the chemical law that when the ingredients for the formation of an insoluble body are present in a mixture this body will always be formed. Medical opinion has for a long time attributed the success attending the use of the lithiated waters to the water and not to the lithium that they contain. A similar error obtains in the case of some more recently introduced substances, lycetol, lysidin, etc. They do not, in the urine, form the very soluble uric acid combinations that they do outside the body. Formic aldehyde being too irritating to be taken internally, Nicolaier then determined to try its amine combination as a substitute. This substance, hexamethylene-tetramine or urotropine, is non-poisonous even in considerable quantities, is unirritating, very soluble in water, and is as good a uric acid solvent as formic aldehyde itself.

The name urotropine was given to it on account of the changes which its administration brought about in the urine. Alkaline and putrid urines, containing mucus in excess, pus and pus organisms, uric acid or amorphous urates, were rapidly restored to a normal appearance and an acid reaction. The urine was sterilized and increased in quantity, and calculi and deposits were dissolved. Hence urotropine is a most valuable resource in all suppurations of the urinary tract, and in all gouty and rheumatic conditions where an active eliminant of uric acid and its salts is indicated. A further valuable property of urotropine is its faculty of combining readily with salicylic acid and forming a soluble combination. A solution containing from ten to fifteen grains each of urotropine and salicylic acid to the fluidounce of water or other suitable vehicle has the further advantage over the salicylates alone in that its taste is not disagreeable. It appears to be far less irritant to the gastric mucous membrane than solutions of salicylic acid usually are, and the combination promises to have a wide range of therapeutic usefulness.—J. A. FLEXNER, M.D., *American Practitioner and News.*

